# Regulation of cyclin T1 during HIV replication and latency establishment in human memory CD4 T cells

**DOI:** 10.1186/s12985-019-1128-6

**Published:** 2019-02-20

**Authors:** Jacob Couturier, Aaron F. Orozco, Hongbing Liu, Sona Budhiraja, Edward B. Siwak, Pramod N. Nehete, K. Jagannadha Sastry, Andrew P. Rice, Dorothy E. Lewis

**Affiliations:** 1 Division of Infectious Diseases, Department of Internal Medicine, University of Texas Health Science Center at Houston, Houston, TX 77030 USA; 2 Department of Molecular Virology & Microbiology, Baylor College of Medicine, Houston, TX USA; 3 Department of Veterinary Sciences, The University of Texas MD Anderson Cancer Center, Bastrop, TX USA; 4 Department of Immunology, The University of Texas MD Anderson Cancer Center, Houston, TX USA

**Keywords:** Cyclin T1, Flow cytometry, HIV latency, HIV replication, HIV reservoirs, Memory CD4 T cells, P-TEFb

## Abstract

**Background:**

The regulatory cyclin, Cyclin T1 (CycT1), is a host factor essential for HIV-1 replication in CD4 T cells and macrophages. The importance of CycT1 and the Positive Transcription Elongation Factor b (P-TEFb) complex for HIV replication is well-established, but regulation of CycT1 expression and protein levels during HIV replication and latency establishment in CD4 T cells is less characterized.

**Methods:**

To better define the regulation of CycT1 levels during HIV replication in CD4 T cells, multiparameter flow cytometry was utilized to study the interaction between HIV replication (intracellular p24) and CycT1 of human peripheral blood memory CD4 T cells infected with HIV in vitro. CycT1 was further examined in CD4 T cells of human lymph nodes.

**Results:**

In activated (CD3+CD28 costimulation) uninfected blood memory CD4 T cells, CycT1 was most significantly upregulated in maximally activated (CD69+CD25+ and HLA.DR+CD38+) cells. In memory CD4 T cells infected with HIV in vitro, two distinct infected populations of p24+CycT1+ and p24+CycT1- cells were observed during 7 days infection, suggestive of different phases of productive HIV replication and subsequent latency establishment. Intriguingly, p24+CycT1- cells were the predominant infected population in activated CD4 T cells, raising the possibility that productively infected cells may transition into latency subsequent to CycT1 downregulation. Additionally, when comparing infected p24+ cells to bystander uninfected p24- cells (after bulk HIV infections), HIV replication significantly increased T cell activation (CD69, CD25, HLA.DR, CD38, and Ki67) without concomitantly increasing CycT1 protein levels, possibly due to hijacking of P-TEFb by the viral Tat protein. Lastly, CycT1 was constitutively expressed at higher levels in lymph node CD4 T cells compared to blood T cells, potentially enhancing latency generation in lymphoid tissues.

**Conclusions:**

CycT1 is most highly upregulated in maximally activated memory CD4 T cells as expected, but may become less associated with T cell activation during HIV replication. The progression into latency may further be predicated by substantial generation of p24+CycT1- cells during HIV replication.

**Electronic supplementary material:**

The online version of this article (10.1186/s12985-019-1128-6) contains supplementary material, which is available to authorized users.

## Background

HIV-1 replication is notable for its strict dependence on host factors, of which Cyclin T1 (CycT1) is one of the most essential for viral RNA transcription in CD4 T cells and macrophages. CycT1 is a regulatory subunit that associates with the cyclin-dependent kinase CDK9 to form the Positive Transcription Elongation Factor b (P-TEFb), an enzymatic complex that phosphorylates the C-terminal domain (CTD) of RNA polymerase II (RNAP II) to initiate mRNA transcriptional elongation [1–3]. Unlike other cyclins (D, E, A, and B), CycT1 is unique in which this cyclin is not known to have significant roles in cell cycle regulation, as CycT1 and CDK9 levels do not oscillate in regulated fashion like the conventional cyclins, but is believed to be more important for T cell activation and differentiation [4–8]. During HIV replication, the viral Tat protein binds to and hijacks P-TEFb, redirecting it to the Transactivation Response Element (TAR) of integrated provirus for mRNA transcription [3, 9–12]. The catalytic activity of P-TEFb is tightly regulated and inhibited by mechanisms such as sequestration with the 7SK snRNP and HEXIM1/2 protein complexes, and the regulation of P-TEFb expression at the protein level involves translational repression of CycT1 by microRNA’s (miRNA) or RNA binding proteins [13–16]. The molecular details and role of P-TEFb and CycT1 for HIV transcription are well defined, but the interaction between CycT1 and HIV replication at single cell levels are less characterized.

Eradication of HIV is impeded by establishment of latent viral reservoirs in CD4 T cells and macrophages. P-TEFb and CycT1 may have important roles for proviral reactivation as studies demonstrate upregulation of these host factors during latency reversal [17–21]. P-TEFb and CycT1 are expressed in resting T cells at very low levels, but substantially increase in naïve and memory CD4 T cells upon activation by stimulants such as CD3/TCR ligation, cytokines (IL2, IL6, and TNFα), and PHA or PMA [4, 22]. During T cell activation, the P-TEFb protein complex becomes catalytically active following phosphorylation of threonine 186 in the T-loop of CDK9 (pCDK9) and facilitates HIV replication, whereas the return of activated CD4 cells to quiescence results in downregulation of pCDK9 and CycT1, concomitant with the establishment of viral latency [23–27]. A more recent study demonstrated that the transition of activated effector CD4 T cells to resting memory CD4 T cells is a critical period for latency establishment [28]. However, other studies show that T cell activation is not strictly required for latency establishment and reactivation. Swiggard et al., Agosto et al., and Dai et al. showed that integration and latency establishment can occur in resting primary CD4 T cells [29–31]. Pace et al. and Chavez et al. also showed that resting CD4 T cells can be latently infected and produce viral proteins, and Cameron et al. and Saleh et al. showed that treatment of resting CD4 T cells with chemokines such as CCL19 and CCL20 promotes latency establishment, conditions in which P-TEFb activation and function is minimal [32–35]. Additionally, Novis et al. showed that stimulation of toll-like receptors (TLRs 1/2) can reactivate latent HIV in memory CD4 T cells without increasing T cell activation, proliferation, and CycT1 expression, whereas activated P-TEFb was more important for viral expression [36]. P-TEFb is critical for HIV post-transcriptional replication, but a better understanding of the dynamics between P-TEFb expression and HIV replication and latency establishment is needed.

In the present study, phenotypic analyses of CycT1 was conducted in human memory CD4 T cells during HIV replication by flow cytometry, which mainly showed substantial amounts of productively infected cells that may be transitioning towards latency following downregulation of CycT1. CycT1 was further upregulated in lymph node CD4 T cells, which may predispose many of these lymphoid cells for infection and latency generation.

## Methods

### Cells and cultures

For purification of memory and naïve CD4 T cells, PBMC were first isolated from buffy coat preparations of de-identified uninfected healthy donors (Gulf Coast Regional Blood Center, Houston, TX) by density-gradient centrifugation with Ficoll-Paque PLUS (GE Healthcare). Memory CD4+CD45RO+CD45RA- or naïve CD4+CD45RO-CD45RA+ T cells were then purified from PBMC by magnetic bead-based EasySep negative selection kits (Stemcell Technologies), and purities were at least 90–95%. Cells were cultured in complete RPMI medium (Hyclone RPMI supplemented with 10% FBS, 2 mM L-glutamine, 0.1 mM MEM nonessential amino acids, 2 mM sodium pyruvate, 25 mM HEPES, and 1X antibiotic-antimycotic) at 37 °C + 5%CO2 prior to experiments. Most experiments involved cultures of 1-2 × 10^6^ uninfected or HIV-infected memory CD4 T cells for 1–7 days in complete RPMI medium (2-3 ml in 24-well plates), without or with stimulation by 1-5 μg/ml coated CD3 + 1-2 μg/ml soluble CD28 mabs and 0.5 μg/ml recombinant IL2 (Biolegend), as specified.

For experiments including antiretroviral compounds, 1 × 10^6^ uninfected or HIV-infected memory CD4 T cells were cultured with indicated stimulants (1 μg/ml coated CD3 or 0.5 μg/ml IL2) for 6 days, with or without either 1 μg/ml AZT, 5 μg/ml Raltegravir, or 0.1 μg/ml Saquinavir (NIH AIDS Reagent Program).

For experiments with lymph node CD4 T cells, mesenteric and inguinal lymph nodes were obtained from de-identified uninfected cadaver donors (National Disease Research Interchange, Philadelphia, PA). Lymph nodes were mechanically minced and incubated in PBS/2%FBS containing 50 μg/ml DNase I and 0.5 mg/ml collagenase II (Sigma) at 37 °C for 1–2 h. Cells were then filtered through 100 μm strainers and washed with PBS/2%FBS. Cells were cultured in complete RPMI medium prior to experiments.

For cell line experiments, J-Lat 6.3 cells were obtained from the NIH AIDS Reagent Program. Cells were cultured in complete RPMI medium (Hyclone RPMI supplemented with 10% FBS, 2 mM L-glutamine, 0.1 mM MEM nonessential amino acids, 2 mM sodium pyruvate, 25 mM HEPES, and 1X antibiotic-antimycotic) at 37 °C + 5%CO2. For PMA treatments, cells were cultured with 0.5 μg/ml PMA for 24 h.

### HIV infections

For infections of memory CD4 T cells, purified cells were infected with virus stocks of R5 strains NSN-SX or SF162 (CFAR Virology Core, Houston, TX). 2 × 10^6^ cells were cultured with virus at MOI of ~ 0.1 for 2 days in complete RPMI medium (1 ml in 48-well plates) and 0.5 μg/ml recombinant IL2. Cells were then washed and cultured for experiments.

For ex vivo infections of lymph node single cell preparations, 1 × 10^6^ cells were cultured with R5 strain virus stocks (SF162) at MOI of ~ 0.1 for 2 days in complete RPMI medium (1 ml in 48-well plates) and 0.5 μg/ml recombinant IL2. Cells were then washed and 1 × 10^6^ cells cultured in complete RPMI medium (3 ml in 24-well plates) and 0.5 μg/ml recombinant IL2 for 6 days.

### Flow cytometry

Flow cytometry data was acquired with a Gallios Flow Cytometer and analyzed with Kaluza software (Beckman-Coulter).

For measurement of surface proteins, cells were washed 2x with 2 ml PBS/2%FBS, then incubated with 1-5 μg/ml fluorochrome-conjugated mabs or matched isotype controls for 30 mins at 4 °C. Antibiodies for surface proteins included CD3 (APCCy7 or APC-AF750), CD4 (PerCPCy5.5 or AF700), CD25 (PE, PECy7, or V450), CD69 (APC), CD38 (PECy7), HLA.DR (AF700 or APCCy7), HLA.ABC (Cy-Chrome), CD62L (APC), or beta7 integrin (FITC) (Biolegend, BD Biosciences, or eBioscience). Cells were then washed 2x with 2 ml PBS/2%FBS. For viability measurements, cells were incubated with 1 μl Zombie Violet fixable dye (Biolegend) for 30 mins at 4 °C prior to surface stainings.

For measurement of CycT1, intracellular HIV p24, p21, or Ki67, cells were first stained for surface proteins as described above, washed, then fixed with 200 μl Cytofix/Cytoperm solution (BD Biosciences). Cells were washed 1x with 2 ml Cytoperm buffer, then incubated with 3 μg/ml CycT1-FITC or the matching Isotype-FITC antibody (goat polyclonal, Santa Cruz Biotechnology), 1 μg/ml p24-PE (monoclonal clone KC57-RD1, Beckman-Coulter), 2 μg/ml p21-AF647 (Santa Cruz Biotechnology), or 1 μg/ml Ki67-AF647 (Biolegend) for 30 mins at 4 °C. Cells were washed 2x with 2 ml Cytoperm buffer, then analyzed with flow cytometer. To confirm specificity of the CycT1-FITC antibody, 1 × 10^6^ activated CD4 T cells were pre-incubated with different amounts of CycT1 blocking peptide (Santa Cruz Biotechnology) for 2 h after cell fixation, followed by addition of CycT1-FITC abs (Additional file 1b). For measurement of phosphorylated-CDK9 (Thr186), cells were washed 2x with 2 ml PBS/2%FBS, then fixed with 4 ml 100% methanol for 12 h at − 20 °C. Cells were washed 3x with PBS/2%FBS, then permeabilized with 1 ml PBS/0.5% Triton X-100 on ice for 10 mins. Cells were washed 2x with PBS/2%FBS, then incubated with 3 μg/ml pCDK9-AF647 (Rabbit IgG1) or matching isotype-AF647 control (Bioss Antibodies) for 2 h at 4 °C. Cells were washed 2x with PBS/2% FBS, then analyzed with flow cytometer.

For cell cycle experiments, 1 × 10^6^ uninfected or HIV-infected memory CD4 T cells were cultured in complete RPMI medium (3 ml in 24-well plates) with 2 μg/ml coated CD3 + 1 μg/ml soluble CD28 mabs and 0.5 μg/ml IL2 for 5 days. Cells were washed 2x with PBS/2%FBS, then fixed with 4 ml 100% methanol for 12 h at − 20 °C. Cells were washed 3x with PBS/2%FBS, then permeabilized with 1 ml PBS/0.5% Triton X-100 on ice for 10 mins. Cells were washed 2x with PBS/2%FBS, then incubated with either 1 μg/ml CycT1-FITC or p24-FITC (Beckman-Coulter) and matching isotype-FITC controls for 1 h at 4 °C. Cells were washed 2x with PBS/2% FBS and incubated 100 μg/ml RNase and 50 μg/ml propidium idodide (Sigma) for 1 h at 4 °C. Cells were then analyzed with flow cytometer.

### Western blots

For western blot analyses, cells were washed with PBS, and lysed in EBCD buffer consisting of 50 mM Tris-HCl (pH 8.0), 120 mM NaCl, 0.5% NP-40, 5 mM DTT, 4 mM MgCl_2_, and protease inhibitor cocktail (Sigma). Equal amounts of proteins were separated by 8% SDS-PAGE, transferred to nitrocellulose membrane, and probed with rabbit anti-human antibodies (1:1000 to 1:5000) for CycT1, CycT2, total CDK9, phosphorylated-CDK9 (Thr186), and actin (Santa Cruz Biotechnology, Bethyl Laboratories, or Cell Signaling Technology). Proteins were visualized via ECL substrate (Pierce).

### Statistics

Statistical analyses were performed using GraphPad Prism. Comparisons between two groups were performed using student’s t-test and *p* values less than 0.05 were considered significant.

## Results

### Significant upregulation of cyclin T1 in activated human memory CD4 T cells

To first characterize CycT1 protein expression of normal uninfected memory CD4 T cells by flow cytometry, memory CD4+CD45RO+ T cells were purified from peripheral blood of healthy donors and activated by CD3+CD28 mabs (costimulation) and IL2 for up to 5 days. Western blot was first used to examine CycT1 expression after 24–72 h costimulation, which showed upregulation during T cell activation (Additional file 1a shows blots representative of two separate experiments). The flow cytometric CycT1 antibody was also tested with CycT1 blocking peptide to confirm specificity with activated naïve and memory CD4 T cells (Additional file 1b).

**Fig. 1:**
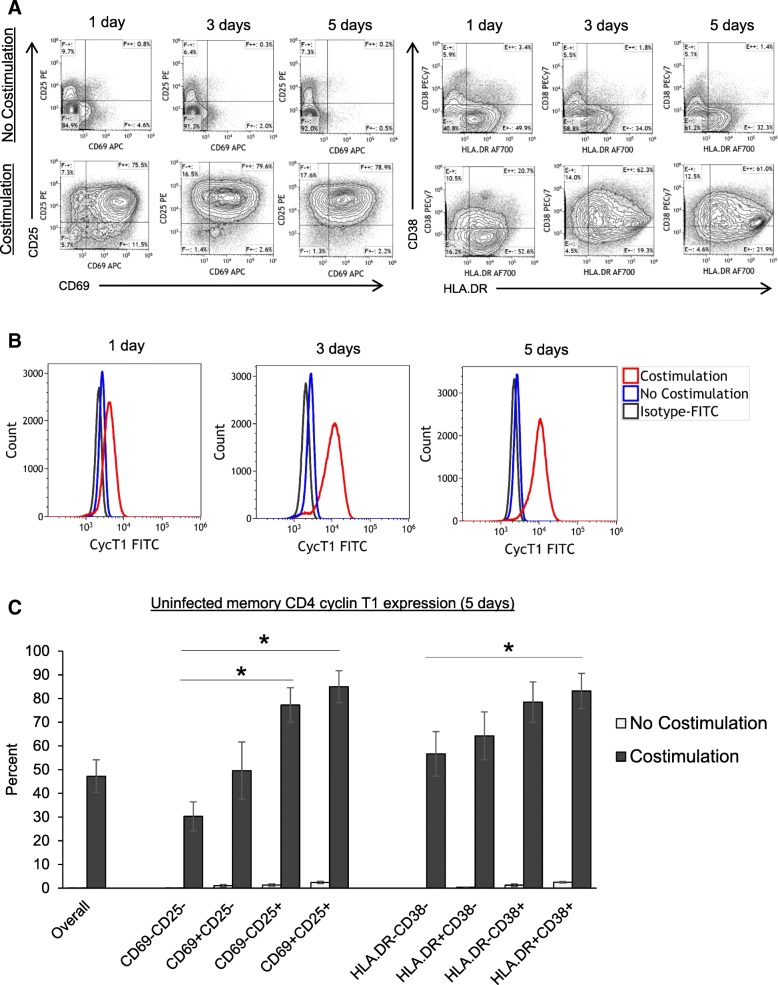
Assessment of CycT1 expression in uninfected memory CD4 T cells during T cell activation. Human CD4+CD45RO+ memory T cells were purified from peripheral blood and cultured without (No Costimulation) or with CD3+CD28 mabs and IL2 (Costimulation) for 1–5 days. Cells were then stained for CycT1, CD69, CD69, HLA.DR, and CD38. **a** Shown are sample flow cytometry dotplots of CD69/CD25 and HLA.DR/CD38 expression of memory CD4 T cells without or with costimulation, and (**b**) overlays of CycT1 expression. **c** Mean ± sem CycT1 expression gated on different CD69/CD25 and HLA.DR/CD38 populations (*p < 0.05, *N* = 3–4)

Next, CycT1 expression was examined in activated memory CD4 T cells. Fig. 1a shows sample flow cytometry dotplots of CD69/CD25 and HLA.DR/CD38 expression during T cell costimulation, and Fig. 1b shows overlays of overall CycT1 expression in non-costimulated or costimulated CD4 T cells. Figure 1c shows mean ± sem CycT1 expression gated on CD69/CD25 and HLA.DR/CD38 populations after 5 days costimulation, in which ~ 50% of memory CD4 T cells overall expressed CycT1, and CycT1 was expressed highest (> 80%) in maximally activated CD69+CD25+ and HLA.DR+CD38+ cells (*N* = 3–4). We also examined CycT1 and T cell activation in the context or small or large cells (Additional file 2), as cell size is associated with T cell activation and HIV latency [37–39]. Additional file 2a shows flow cytometry dotplots of CycT1 expression (based on Isotype-FITC controls) gated on overall, small, or large cells, and without or with CD3+CD28 costimulation. Additional file 2b shows mean ± sem CycT1, CD69+CD25+, and HLA.DR+CD38+ expression gated on overall, small, or large cells. CycT1 levels were mostly similar amongst overall, small, and large cells (~30–50%), whereas CD69+CD25+ and HLA.DR+CD38+ expression was higher in large compared to small cells (*p* < 0.05, *N* = 5).

**Fig. 2:**
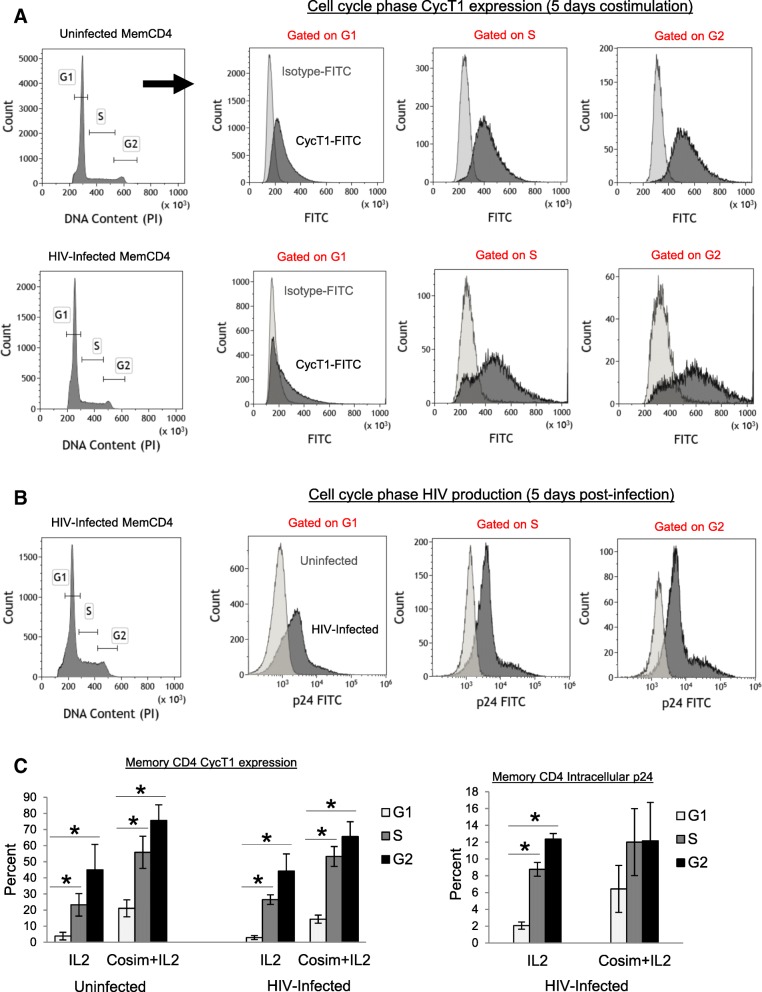
CycT1 expression and HIV production during cell cycle progression of memory CD4 T cells. Memory CD4 T cells were uninfected or HIV-infected (R5 strain SF162) in IL2 medium for 2 days, washed, and cultured for 5 days with CD3+CD28 costimulation and IL2 or IL2 alone. Cells were then stained with propidium iodide for DNA content in conjunction with either CycT1-FITC or p24-FITC abs. **a** Sample cell cycle distributions and CycT1 expression in G1, S, or G2 phases of uninfected and HIV-infected memory CD4 T cells after 5 days costimulation. **b** Sample cell cycle distributions and intracellular p24 levels in G1, S, or G2 phases of HIV-infected memory CD4 T cells after 5 days costimulation. **c** Mean ± sem CycT1 and p24 levels during cell cycle progression of memory CD4 T cells (*p < 0.05, *N* = 3)

Lastly, CycT1 and HIV replication were examined during cell cycle progression of memory CD4 T cells during culture with IL2 alone or CD3+CD28 costimulation for 5 days (Fig. 2). Unlike conventional cyclins, CycT1 is unknown to regulate cell cycle progression and CycT1 levels do not oscillate in coordinated fashion during T cell activation and proliferation, although CycT1 expression patterns specifically in G1, S, and G2 phases of T cells have not been reported. Fig. 2a shows sample CycT1-FITC and Isotype-FITC levels gated on G1, S, or G2 phases of uninfected or HIV-infected memory CD4 T cells after 5 days costimulation, and Fig. 2b shows HIV intracellular p24 levels gated on G1, S, or G2 phases. As expected, CycT1 and p24 levels were generally higher in S and G2 phases compared to G1 (Fig. 2c shows mean ± sem CycT1 and p24 expression, *p* < 0.05, *N* = 3). Altogether, these data show that CycT1 expression is strongly associated with T cell activation status, with highest levels in maximally activated (CD69+CD25+ and HLA.DR+CD38+) memory CD4 T cells.

### Potential transition from productive HIV replication to latency following downregulation of CycT1

**Fig. 3:**
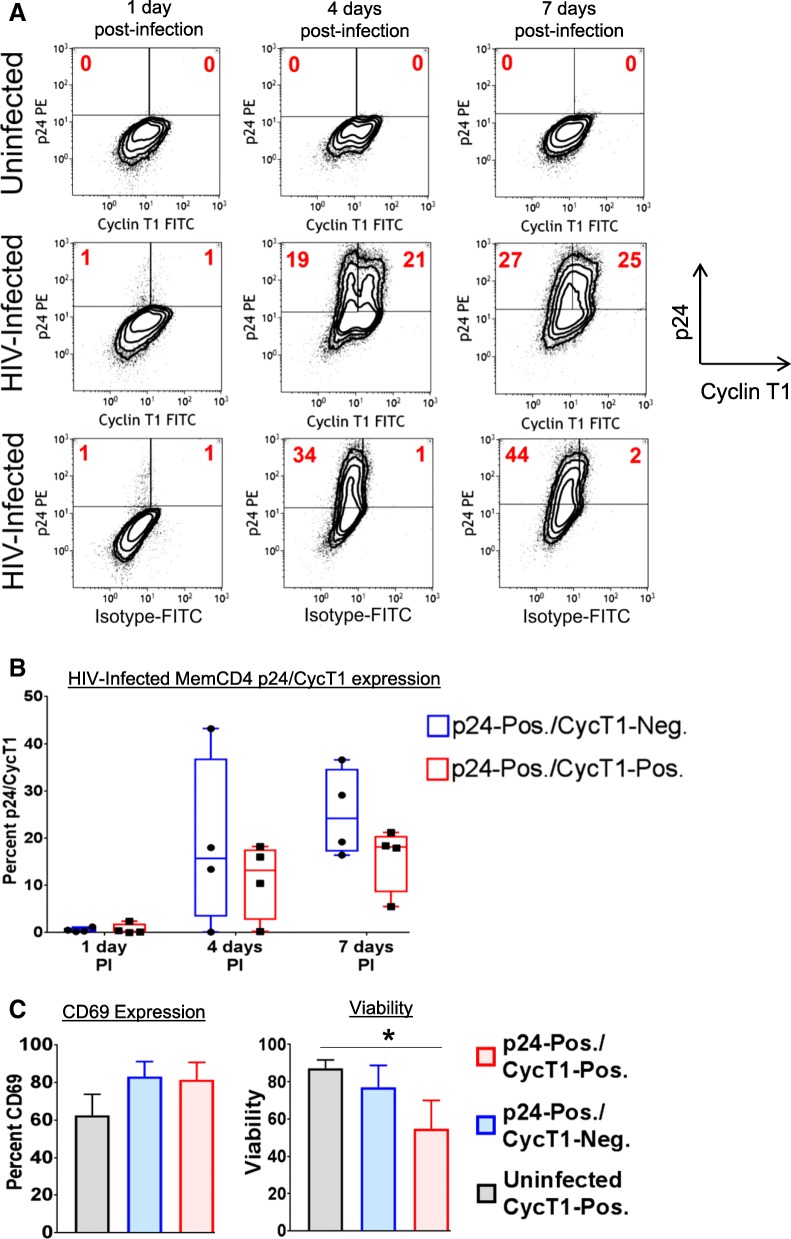
Generation of p24+CycT1- and p24+CycT1+ cells during HIV replication in memory CD4 T cells. Memory CD4 T cells were purified from peripheral blood, then uninfected or infected with HIV (R5 strain NSN-SX) in IL2 medium for 2 days. Cells were washed and cultured with CD3+CD28 mabs and IL2 for 7 days. Cells were then stained for CycT1 and intracellular p24. **a**-**b** Shown are sample dotplots and mean ± sem p24/CycT1 expression during HIV replication (*N* = 3–4). **c** Activation (CD69 expression) and viability levels of uninfected CycT1+ and infected p24+CycT1- and p24+CycT1+ memory CD4 T cells (*p < 0.05)

To next determine phenotypic profiles of CycT1 expression during HIV replication, memory CD4 T cells were infected with HIV (R5 strain NSN-SX) for 2 days in IL2 medium, washed, then cultured with CD3+CD28 mabs and IL2 for up to 7 days. After 4–7 days post-infection, two distinct populations of p24+CycT1- and p24+CycT1+ cells were observed (Fig. 3a shows sample p24/CycT1 dotplots). By 7 days post-infection, ~25% of infected memory CD4 T cells were p24+CycT1-, and ~16% were p24+CycT1+ (Fig. 3b, *N* = 3). These two p24/CycT1 populations likely depict the progression of infected CD4 T cells from productive replication (p24+CycT1+) towards latency establishment consequent to CycT1 downregulation (p24+CycT1-) [25]. T cell activation levels (CD69) were similar (~ 80%) between p24+CycT1- and p24+CycT1+ cells, while viability was lower in p24+CycT1+ cells (Fig. 3c). However, the p24+CycT1- cells are likely not completely devoid of CycT1 protein (due to sensitivity limits of flow cytometry for nuclear proteins), but suggest significant downregulation of CycT1 protein compared to p24+CycT1+ cells.

**Fig. 4:**
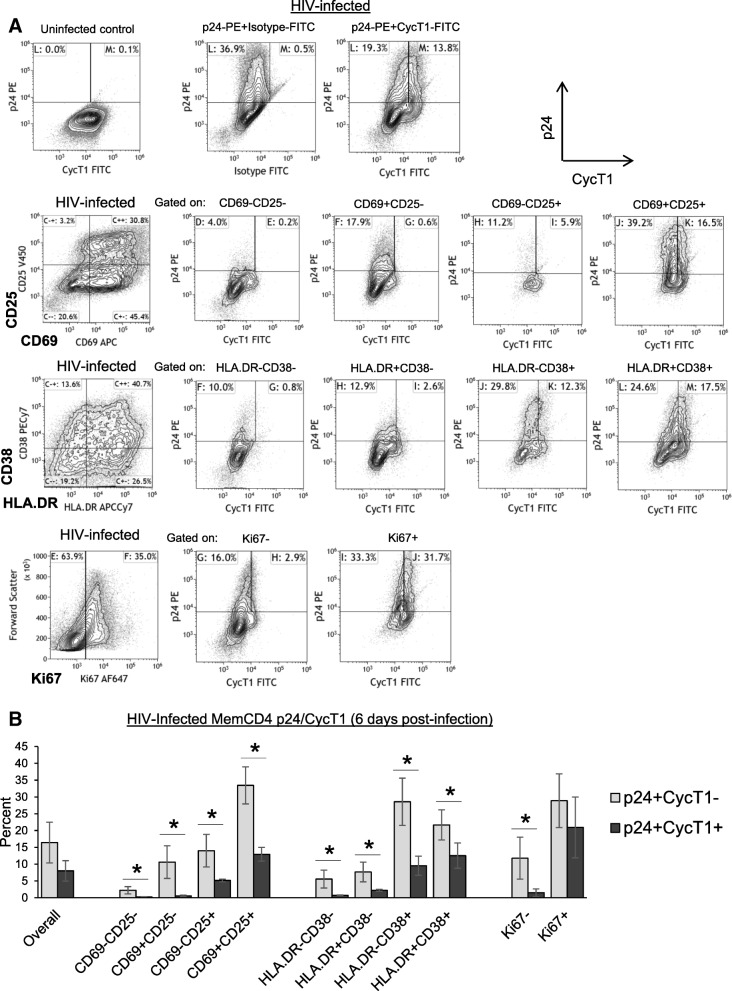
Progression of HIV-infected p24+CycT1- cells into latency following activation of memory CD4 T cells. Memory CD4 T cells were purified from peripheral blood, then uninfected or infected with HIV (R5 strain SF162) in IL2 medium for 2 days. Cell were washed and cultured with CD3+CD28 mabs and IL2 for 6 days. Cells were then stained for CycT1 and intracellular p24 in conjunction with either CD69/CD25, HLA.DR/CD38, or Ki67. **a** Shown are sample dotplots and (**b**) mean ± sem p24+CycT1- and p24+CycT1+ cells gated on CD25/CD69, HLA.DR/CD38, and Ki67 populations of HIV-infected memory CD4 T cells (*p < 0.05, *N* = 3–4)

CycT1 expression and P-TEFb activation are strongly induced upon T cell activation [23, 24]. To better define p24/CycT1 distribution with respect to activation status of HIV-infected CD4 T cells, p24 and CycT1 were examined in conjunction with CD69/CD25, HLA.DR/CD38, or Ki67 (Fig. 4). Figure 4a and b show sample flow cytometry dotplots and mean ± sem p24+CycT1+ and p24+CycT1- memory CD4 T cells gated on CD69/CD25, HLA.DR/CD38, or Ki67 populations after CD3+CD28 costimulation and IL2 for 6 days (*N* = 3–4). Both p24+CycT1+ and p24+CycT1- cells were increased in more activated cells, but in virtually all cell populations, p24+CycT1- cells were the predominant infected subset, even in maximally activated (CD69+CD25+ and HLA.DR+CD38+) cells (*p* < 0.05 comparing p24+CycT1+ to p24+CycT1- cells), indicating potential latency establishment may be higher in more activated CD4 T cells. The dichotomy between p24+CycT1+ and p24+CycT1- cells in CD69/CD25 populations was more pronounced if CD4 T cells were cultured in more modest stimulation conditions that were not as “activating” such as 0.5 μg/ml CD3 mabs alone or 0.1 μg/ml IL2 alone for 6 days, which induce CycT1 expression less compared to CD3+CD28 costimulation, perhaps favoring latency establishment more than productive replication (Additional file 3, *N* = 3–4). Altogether, these data are consistent with the idea that CycT1 levels are a major determinant driving infected CD4 T cells from active replication towards latency establishment [25].

### Hijacking of CycT1 during HIV replication

**Fig. 5:**
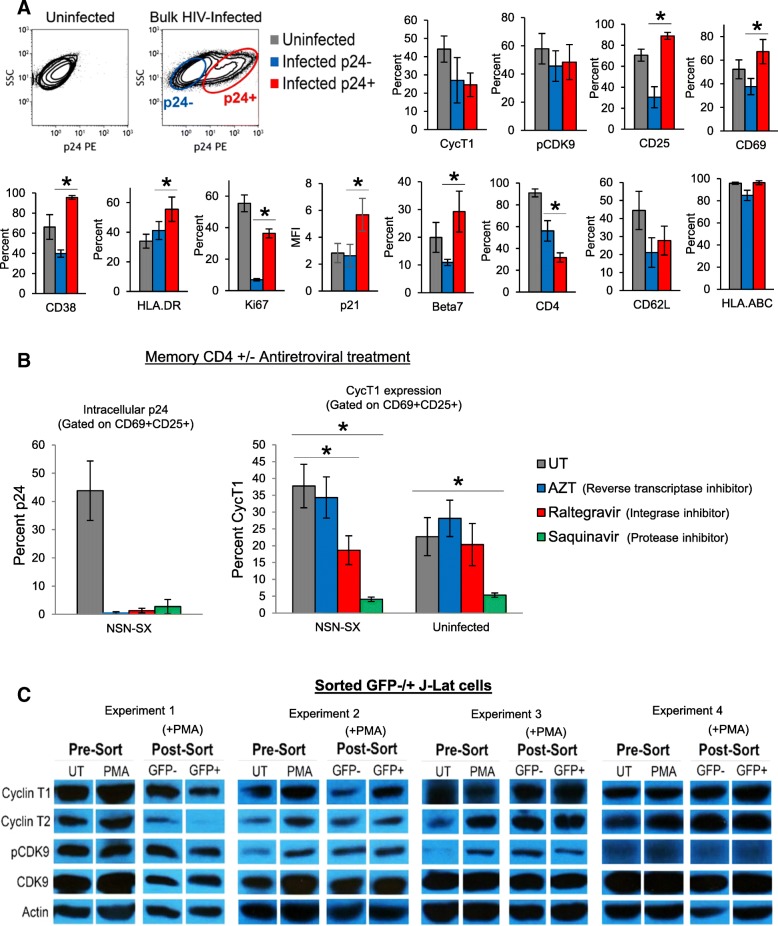
Minimal interactions between HIV replication and CycT1 levels in memory CD4 T cells. **a** Memory CD4 T cells were purified from peripheral blood, then uninfected or infected with HIV (R5 strain NSN-SX) in IL2 medium for 2 days. Cells were washed and cultured with CD3+CD28 mabs and IL2 for 6 days. Cells were then stained for intracellular p24 in conjunction with either CycT1, phosphorylated-CDK9 (Thr186), CD25, CD69, CD38, HLA.DR, Ki67, p21, beta7 integrin, CD4, CD62L, or HLA.ABC. Shown are sample dotplots of uninfected and HIV-infected bystander p24- and infected p24+ cells, and mean ± sem expression of proteins in uninfected and infected bystander p24- and infected p24+ cells (*p < 0.05 comparing p24- to p24+ cells, *N* = 3–5). **b** Reduction of HIV replication concomitant with CycT1 reduction in memory CD4 T cells by raltegravir. HIV-infected memory CD4 T cells (5 days post-infection) were treated with AZT, raltegravir, or saquinavir for 3 days, then stained for p24, CycT1, CD25, and CD69. Shown are mean ± sem p24 and CycT1 levels gated on CD69+CD25+ cells (*p < 0.05, *N* = 3). **c** Comparison of P-TEFb protein levels between GFP- and GFP+ J-Lat6.3 cell lines by western blot. Shown are western blots of CycT1, CycT2, pCDK9, and total CDK9 in non-sorted or sorted (following 24 h PMA treatment) GFP- or GFP+ J-Lat cells

As CycT1 and P-TEFb function are highly associated with T cell activation, we next determined if HIV replication also increases CycT1 expression, as HIV replication is known to further potentiate T cell activation. Memory CD4 T cells were infected with HIV (R5 strain NSN-SX) for 6 days, and surface and intracellular proteins were compared amongst bulk uninfected, infected bystander p24-, and infected p24+ cells (Fig. 5a). We examined CycT1 and phosphorylated-CDK9 (Thr186 pCDK9 - a marker of activated P-TEFb), activation markers (CD25, CD69, CD38, HLA.DR, and Ki67), and other proteins regulated by HIV infection for comparison (CD4, p21, beta7 integrin, CD62L, and HLA.ABC). As expected, the activation markers CD25, CD69, CD38, HLA.DR, and Ki67 were significantly increased in infected p24+ cells compared to bystander p24- cells, whereas CD4 expression was decreased (*p* < 0.05, *N* = 3–5). Additionally, intracellular p21 and surface Beta7 integrin were also increased by HIV replication. Despite the significant increases of multiple activation markers, CycT1 and pCDK9 levels remained similar between p24+ and p24- cells, indicating marginal effects of HIV replication upon P-TEFb function and stability, or the likely hijacking of P-TEFb proteins by viral Tat during HIV replication.

To next determine if inhibiting HIV replication affects CycT1 levels, HIV-infected or uninfected memory CD4 T cells were treated with antiretroviral drugs (AZT, raltegravir, or saquinavir) for 3 days, then examined for CycT1 expression (Fig. 5b). All three drugs substantially reduced HIV production as expected, but in maximally activated (CD69+CD25+) cells, only the integrase inhibitor raltegravir decreased CycT1 levels concomitant with p24 reductions (*p* < 0.05), and without affecting CycT1 expression in control uninfected cells (which was observed with saquinavir).

We lastly GFP-sorted HIV latently infected J-Lat6.3 cells (after 24 h PMA treatment) and conducted western blots for CycT1 and other P-TEFb component proteins including CycT2, CDK9 and pCDK9 (Fig. 5c). Western blots of four experiments showed that CycT1 and activated P-TEFb (pCDK9) levels were variable or mostly similar between GFP- and GFP+ cells, consistent with the data in Fig. 5a and b, suggesting overall that HIV replication may not significantly affect CycT1 expression.

### Cyclin T1 as a host susceptibility factor in lymph node CD4 T cells

We lastly studied CycT1 regulation in CD4 cells in lymph nodes, as these cells are major HIV reservoirs [40–43]. The underlying molecular mechanisms which make lymph node CD4 T cells more susceptible to HIV infection and latency are unclear, but may be due to higher states of activation [43]. Additionally, lymph node T cells constitutively express CDK9 and CycT1 (previously reported by immunohistochemistry), and P-TEFb and CycT1 are important regulators of Tfh and Th1 differentiation [7, 8, 44].

**Fig. 6:**
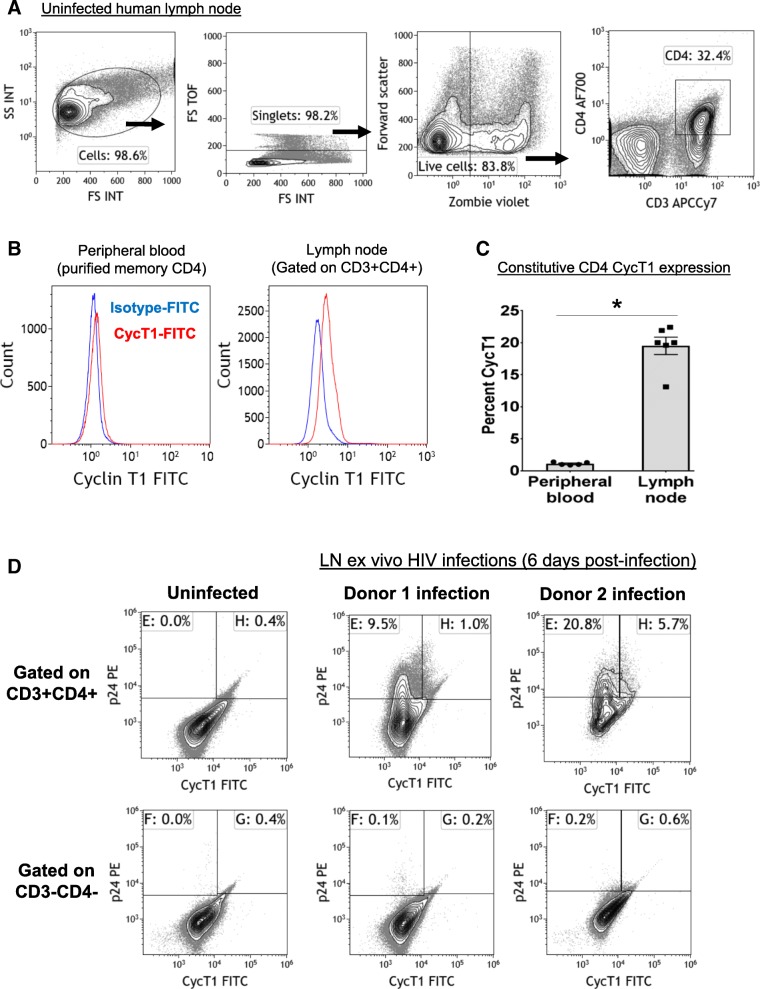
Upregulation of CycT1 in lymph node CD4 T cells. **a**-**c** Constitutive expression levels of CycT1 in blood or lymph node CD4 T cells of uninfected donors. CD4+CD45RO+ memory T cells were purified from peripheral blood, or lymph nodes were processed into single cells. Cells were then stained for CD3, CD4, and CycT1. Shown are (**a**) gating scheme dotplots and (**b**) overlays of CycT1 expression compared to isotype control, and (**c**) mean ± sem CycT1 expression in total CD4 T cells (*p < 0.05, *N* = 5–6). **d** p24/CycT1 expression in lymph node CD4 T cells infected with HIV ex vivo. Lymph nodes of two uninfected donors were processed into single cells, infected with HIV (R5 strain SF162) in IL2 medium for 2 days, washed, and cultured in IL2 medium for 6 days. Cells were then stained for CD3, CD4, CycT1, and p24. Shown are p24/CycT1 dotplots of an uninfected and two infected donors gated on CD3-CD4- or CD3+CD4+ cells

CycT1 expression was examined in lymph nodes of uninfected healthy donors (Fig. 6). Lymph nodes were processed into single cells and CycT1 examined gated on CD3+CD4+ T cells, and memory CD4+CD45RO+ T cells purified from peripheral blood were examined for comparison. Figure 6a and b show a sample flow cytometry gating scheme and CycT1 histogram overlays. Compared to blood memory CD4 T cells which constitutively expressed less than 2% CycT1, the constitutive expression levels of CycT1 in lymph node CD4 T cells were significantly higher at ~ 20% (Fig. 6c, *p* < 0.05, *N* = 5–6). Lastly, lymph nodes from two healthy donors were processed into single cells and infected with HIV (R5 strain SF162) in IL2 medium for 2 days (Fig. 6d). Cells were washed and cultured in IL2 medium for 6 days. Cells were then examined for p24/CycT1 expression gated on CD3-CD4- or CD3+CD4+ cells. Figure 6d shows dotplots of p24+CycT1- and p24+CycT1+ cells gated on CD3-CD4- or CD3+CD4+ cells of the two donors, showing that p24+CycT1- cells of CD3+CD4+ T cells were predominantly higher than p24+CycT1+ cells. These data suggest that CycT1 may be an important host factor that increases the permissiveness of lymph node CD4 T cells for HIV infection and latency establishment.

## Discussion

Mechanisms of HIV latency establishment remain incompletely understood. Latency mainly occurs consequent to the return of infected activated CD4 T cells to a resting state (post-integration latency) [45, 46]. CycT1 is a host factor upregulated during T cell activation and critical for HIV post-transcriptional replication, but its role during latency establishment is less clear. The present study suggests that the return of infected activated CD4 T cells to quiescence is preceded by downregulation of CycT1 expression and P-TEFb function, significantly restricting HIV replication and promoting latency establishment.

T cell activation by CD3+CD28 costimulation significantly upregulated CycT1 in memory CD4 T cells (Fig. 1), consistent with previous reports [23, 26]. However, examination of HIV replication via intracellular p24 in conjunction with CycT1 showed two distinct populations of p24+CycT1- and p24+CycT1+ cells (Fig. 3). Both subsets were progressively increased as cells became more activated (comparing from CD69-CD25- to CD69+CD25+, from HLA.DR-CD38- to HLA.DR+CD38+, or from Ki67- to Ki67+) (Fig. 4). Whereas the p24+CycT1+ cells denote optimal HIV replication in maximally activated CD4 T cells, the p24+CycT1- cells most likely represent productively infected cells destined for latency subsequent to CycT1 downregulation, and these p24+CycT1- cells typically remained the predominant infected cell type in most activated CD4 populations. Budhiraja et al. previously showed that CycT1 and activated P-TEFb are present at low levels in human resting memory CD4 T cells, and that following HIV infections of activated CD4 T cells (which had high levels of CycT1 and P-TEFb), the subsequent establishment of viral latency was associated with downregulation of CycT1 and P-TEFb as cells returned to resting states [25, 47]. The culture of infected CD4 T cells in more modest or low-level stimulation conditions such as IL2 alone or anti-CD3 mabs alone shown in Additional file 3 appeared to further increase the proportion of p24+CycT1- cells relative to p24+CycT1+ cells, even in maximally activated CD69+CD25+ cells. Such generation of p24+CycT1- cells may be consistent with scenarios in which other modest forms of stimulation, including homeostatic cytokines (IL2, IL7, and IL15), chemokines (CCL19 and CCL20) or TLR 1/2 ligands, promote HIV latency establishment and induce low-level viral replication in CD4 T cells without significant induction of T cell activation and CycT1 expression [29–36]. Thus, HIV latency may be largely facilitated by downregulation of CycT1 as activated CD4 T cells return to quiescence.

Despite the clear effects of HIV replication on some CD4 T cell functions shown in Fig. 5a, such as increasing activation markers (CD25, CD69, HLA.DR, CD38, and Ki67) and decreasing CD4 expression, CycT1 levels remained mostly unaffected by HIV replication when comparing infected p24+ cells to bystander p24- cells. Additionally, inhibition of HIV replication with antiretroviral drugs in activated CD4 T cells resulted in concomitant but modest downregulation of CycT1 only with the integrase inhibitor raltegravir (Fig. 5b), and western blots of J-Lat cells showed mostly comparable levels of CycT1 and P-TEFb proteins between sorted GFP- and GFP+ cells (Fig. 5c). Possible reasons for these results may include reaching a threshold for maximal expression of CycT1 in p24+ cells in conjunction with hijacking of CycT1 and P-TEFb function during HIV replication, as sequestration of P-TEFb for proviral transcription is a major function of the viral Tat protein. Conversely, a lack of CycT1 downregulation in p24+ cells compared to p24- cells may be due to protein stability, as previous reports have described a relatively long half-life of CycT1 in cell lines and activated human CD4 T cells [23, 25, 48]. The upregulation of p21 in p24+ cells (Fig. 5a) is consistent with a recent report by Guha et al. who showed significantly higher levels of p21 in infected (NL4.3-EGFP+) primary human blood CD4 T cells compared to EGFP- cells [49]. p21 is a cyclin-dependent kinase inhibitor upregulated in CD4 T cells of elite controllers and functions to suppress HIV reverse transcription by inhibiting CDK2-dependent phosphorylation [50, 51]. Interestingly, p21 is also reported to interact with CycT1 and reduce P-TEFb activation, which may have been a factor contributing to the lack of CycT1 upregulation in infected p24+ cells observed in our study [50]. Long-term HIV persistence likely requires a homeostatic balance and moderated levels of host factors such as P-TEFb that allows for optimal viral replication, but also maximizes host cell survival.

Some important limitations of our study include the sensitivity level of flow cytometry for nuclear proteins, the lack of examination of CycT1 and HIV mRNA in conjunction with protein levels, and further confirmation that the p24+CycT1- cells do indeed progress to latency. Although immunofluorescence can be more sensitive than western blot, technical aspects such as fixatives, antibodies and fluorochomes can influence protein detection, and we emphasize that the p24+CycT1- cells likely have some minimal amounts of CycT1 protein. Additionally, CycT1 mRNA and protein levels do not always strictly correlate and can differ with respect to expression, stability, and half-life. For example, Marshall et al. showed with human blood T cells that PHA upregulates both CycT1 mRNA and protein due to CycT1 mRNA stability, whereas PMA upregulated CycT1 protein due to enhanced protein stability without increasing CycT1 mRNA [23]. Clarifying the kinetics of CycT1 mRNA and protein during HIV replication may best be addressed with newer technologies such as PrimeFlow, which would permit simultaneous measurement of both CycT1 and HIV mRNA and protein, and in conjunction with other host proteins [52–54]. Lastly, HIV latency is best studied by infection of activated CD4 T cells and return to a quiescent and resting state, followed by proviral reactivation of purified resting CD4 T cells. Although sorted p24+CycT1- cells cannot be cultured due to cell fixation requirements, HIV DNA could still be examined. Additionally, CycT1 could be examined during infection with HIV Duo-Fluo constructs which indicate latent and productive infection with mCherry and EGFP inserts [34, 55].

## Conclusions

CycT1 is significantly associated with activation levels of uninfected memory CD4 T cells. However, increases of T cell activation during HIV replication do not necessarily also increase CycT1 expression, possibly due to hijacking of P-TEFb for viral replication. During HIV replication in CD4 T cells, there is substantial accumulation of infected p24+CycT1- cells relative to p24+CycT1+ cells, reflecting the potential transition from productive replication to latency establishment following downregulation of CycT1. CycT1 may further be a significant host factor promoting HIV infection and latency in lymph node CD4 T cells.

## Additional files

Additional file 1: Western blot measurement of CycT1 and validation of CycT1 flow cytometric antibody in human memory CD4 T cells. (A) CD4+CD45RO+ memory T cells were purified from peripheral blood and cultured with 5 μg/ml coated CD3 + 2 μg/ml soluble CD28 mabs (costimulation) for up to 72 h. Cell lysates were examined for CycT1 protein levels (shown is a western blot representative of two separate experiments). (B) CD4+CD45RO- naïve and CD4+CD45RO+ memory T cells were purified from blood and cultured with CD3+CD28 mabs for 72 h. Cells were harvested and pre-incubated with CycT1 blocking peptide for 2 h prior to staining with CycT1-FITC antibody. Shown are CycT1 levels representative of two separate experiments. (PPTX 665 kb)

Additional file 2: Cyclin T1 expression in small and large memory CD4 T cells during T cell activation. Human CD4+CD45RO+ memory T cells were purified from peripheral blood and cultured without (No Costimulation) or with CD3 + CD28 mabs and IL2 (Costimulation) for 5 days, then stained for CycT1, CD69, CD25, HLA.DR, and CD38. (A) Shown are sample Isotype-FITC or CycT1-FITC dotplots gated on overall, small, or large cells, and (B) mean ± sem CycT1, CD69+CD25+, or HLA.DR+CD38+ expression (*N* = 5). (PPTX 595 kb)

Additional file 3: Generation of p24/CycT1 cells during HIV replication in modestly stimulated memory CD4 T cells. Memory CD4 T cells were purified from peripheral blood and uninfected or infected with HIV (R5 strain SF162) in IL2 medium for 2 days. Cells were washed and cultured with either 0.5 μg/ml CD3 mabs alone or 0.1 μg/ml IL2 alone for 6 days. Cells were then stained for p24, CycT1, CD69, and CD25. (A-B) Shown are sample dotplots and mean ± sem p24+CycT1- and p24+CycT1+ cells gated on CD69/CD25 populations (*p < 0.05, *N* = 3). (PPTX 443 kb)

## Abbreviations

CDK9: Cyclin-dependent kinase 9
MFI: Mean fluorescence intensity
PMA/IO: Phorbol 12-myristate 13-acetate / ionomycin
P-TEFb: Positive transcription elongation factor b
TLR: Toll-like receptor
