# Supplementary figures and images for: Regulation of cyclin T1 during HIV replication and latency establishment in human memory CD4 T cells
Source: Virol J. 2019 Feb 20;16:22. doi: 10.1186/s12985-019-1128-6 (PMC6381639; doi:10.1186/s12985-019-1128-6)

## Slide 1
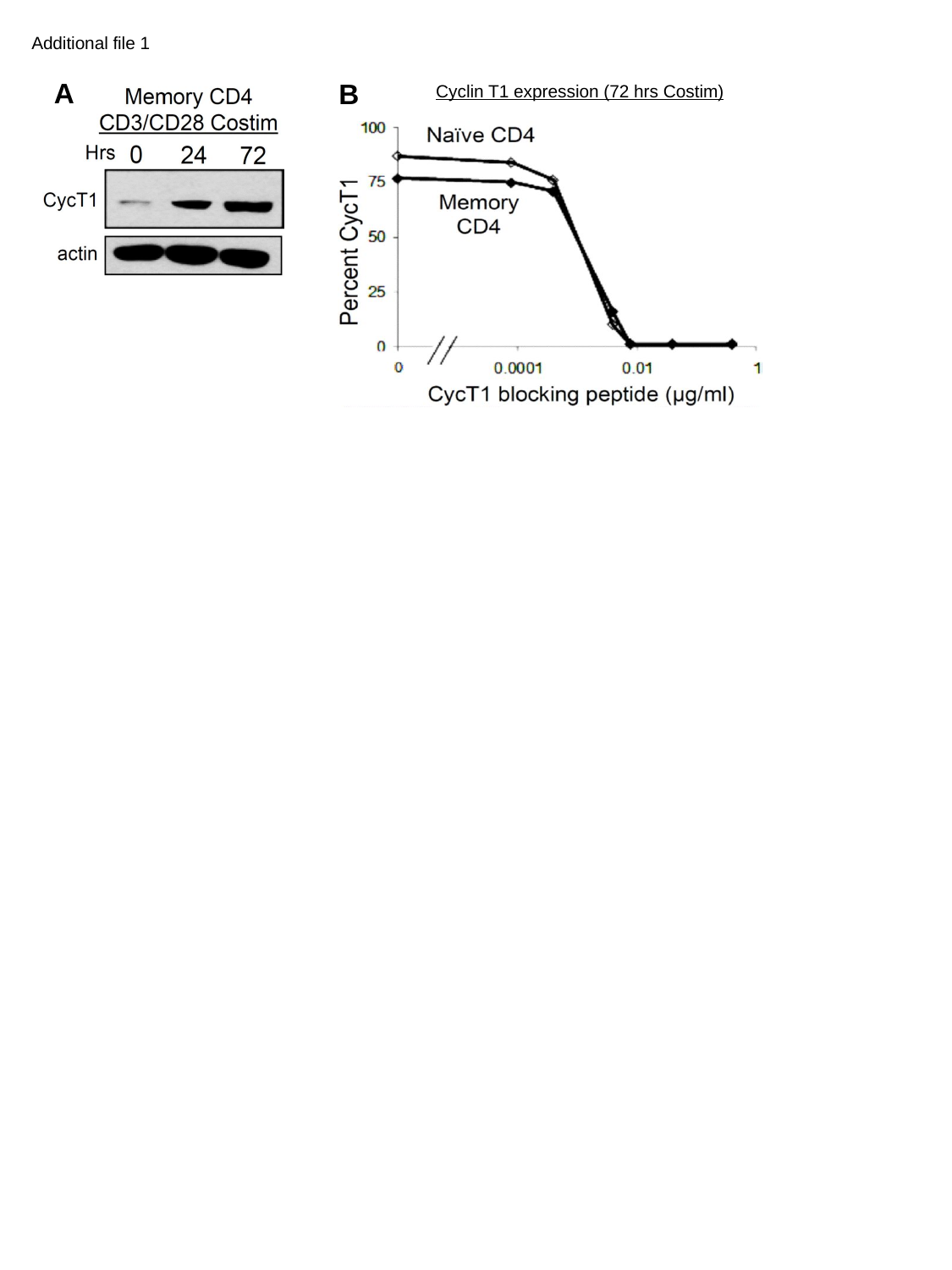

Additional file 1
A
B
Cyclin T1 expression (72 hrs Costim)

Supplement: Supplementary file 1 — Western blot measurement of CycT1 and validation of CycT1 flow cytometric antibody in human memory CD4 T cells. (A) CD4+CD45RO+ memory T cells were purified from peripheral blood and cultured with 5 μg/ml coated CD3 + 2 μg/ml soluble CD28 mabs (costimulation) for up to 72 h. Cell lysates were examined for CycT1 protein levels (shown is a western blot representative of two separate experiments). (B) CD4+CD45RO- naïve and CD4+CD45RO+ memory T cells were purified from blood and cultured with CD3+CD28 mabs for 72 h. Cells were harvested and pre-incubated with CycT1 blocking peptide for 2 h prior to staining with CycT1-FITC antibody. Shown are CycT1 levels representative of two separate experiments. (PPTX 665 kb) [file 12985_2019_1128_MOESM1_ESM.pptx]
