# Supplementary material for: Regulation of cyclin T1 during HIV replication and latency establishment in human memory CD4 T cells
Source: Virol J. 2019 Feb 20;16:22. doi: 10.1186/s12985-019-1128-6 (PMC6381639; doi:10.1186/s12985-019-1128-6)
Supplement: Supplementary file 2 — Cyclin T1 expression in small and large memory CD4 T cells during T cell activation. Human CD4+CD45RO+ memory T cells were purified from peripheral blood and cultured without (No Costimulation) or with CD3 + CD28 mabs and IL2 (Costimulation) for 5 days, then stained for CycT1, CD69, CD25, HLA.DR, and CD38. (A) Shown are sample Isotype-FITC or CycT1-FITC dotplots gated on overall, small, or large cells, and (B) mean ± sem CycT1, CD69+CD25+, or HLA.DR+CD38+ expression (N = 5). (PPTX 595 kb) [file 12985_2019_1128_MOESM2_ESM.pptx]

## Slide 1
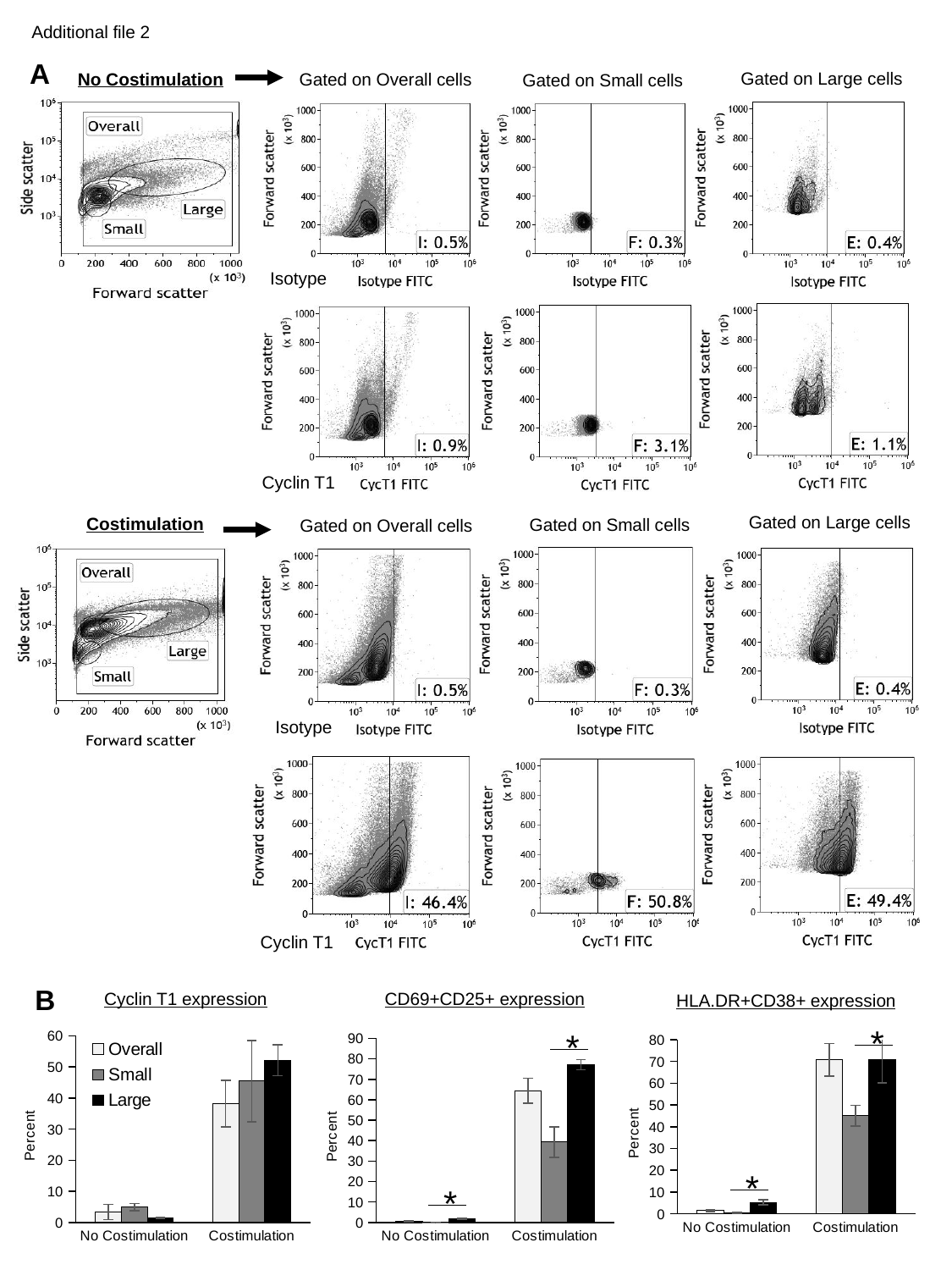

Additional file 2
A
Gated on Large cells
No Costimulation
Gated on Overall cells
Gated on Small cells
Isotype
Cyclin T1
Gated on Large cells
Costimulation
Gated on Small cells
Gated on Overall cells
Isotype
Cyclin T1
B
Cyclin T1 expression
CD69+CD25+ expression
HLA.DR+CD38+ expression
*
### Chart
| Category | Overall | Small | Large |
|---|---|---|---|
| No Costimulation | 3.3199999999999994 | 4.9 | 1.52 |
| Costimulation | 38.260000000000005 | 45.42 | 52.17999999999999 |*
### Chart
| Category | Overall | Small | Large |
|---|---|---|---|
| No Costimulation | 1.46 | 0.3400000000000001 | 5.28 |
| Costimulation | 70.76000000000002 | 45.04 | 71.0 |
### Chart
| Category | Overall | Small | Large |
|---|---|---|---|
| No Costimulation | 0.58 | 0.12000000000000002 | 1.78 |
| Costimulation | 64.4 | 39.26 | 77.16 |*
*
