# Supplementary material for: Regulation of cyclin T1 during HIV replication and latency establishment in human memory CD4 T cells
Source: Virol J. 2019 Feb 20;16:22. doi: 10.1186/s12985-019-1128-6 (PMC6381639; doi:10.1186/s12985-019-1128-6)
Supplement: Supplementary file 3 — Generation of p24/CycT1 cells during HIV replication in modestly stimulated memory CD4 T cells. Memory CD4 T cells were purified from peripheral blood and uninfected or infected with HIV (R5 strain SF162) in IL2 medium for 2 days. Cells were washed and cultured with either 0.5 μg/ml CD3 mabs alone or 0.1 μg/ml IL2 alone for 6 days. Cells were then stained for p24, CycT1, CD69, and CD25. (A-B) Shown are sample dotplots and mean ± sem p24+CycT1- and p24+CycT1+ cells gated on CD69/CD25 populations (*p < 0.05, N = 3). (PPTX 443 kb) [file 12985_2019_1128_MOESM3_ESM.pptx]

## Slide 1
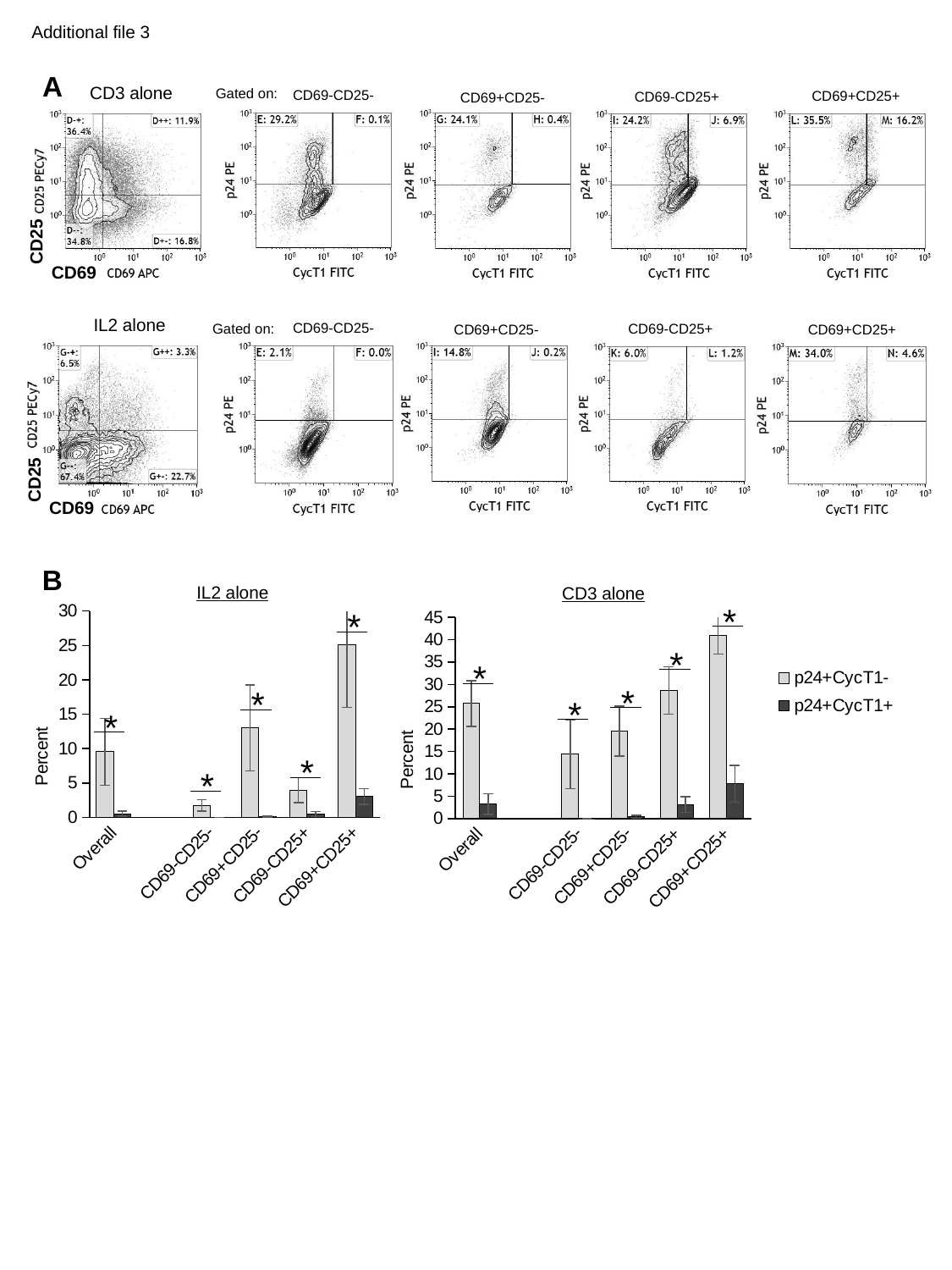

Additional file 3
A
CD3 alone
Gated on:
CD69-CD25-
CD69+CD25+
CD69-CD25+
CD69+CD25-
CD25
CD69
IL2 alone
CD69-CD25-
Gated on:
CD69-CD25+
CD69+CD25-
CD69+CD25+
CD25
CD69
B
IL2 alone
CD3 alone
### Chart
| Category | p24+CycT1- | p24+CycT1+ |
|---|---|---|
| Overall | 9.533333333333333 | 0.5333333333333333 |
| | None | None |
| CD69-CD25- | 1.7333333333333332 | 0.0 |
| CD69+CD25- | 13.0 | 0.13333333333333333 |
| CD69-CD25+ | 3.9333333333333336 | 0.46666666666666673 |
| CD69+CD25+ | 25.133333333333336 | 3.033333333333333 |
### Chart
| Category | p24+CycT1- | p24+CycT1+ |
|---|---|---|
| Overall | 25.766666666666666 | 3.233333333333334 |
| | None | None |
| CD69-CD25- | 14.4 | 0.03333333333333333 |
| CD69+CD25- | 19.566666666666666 | 0.43333333333333335 |
| CD69-CD25+ | 28.666666666666668 | 3.1666666666666665 |
| CD69+CD25+ | 41.0 | 7.766666666666667 |*
*
*
*
*
*
*
*
*
*
